# Spotted fever group *Rickettsia* infection kinetics and cutaneous host response during single tick feeding events

**DOI:** 10.1371/journal.ppat.1014626

**Published:** 2026-09-28

**Authors:** Chanakan Suwanbongkot, Ingeborg M. Langohr, Killian Z. Brewer, Robert A. Barrington, Kevin R. Macaluso

**Affiliations:** 1 Department of Microbiology and Immunology, Frederick P. Whiddon College of Medicine, University of South Alabama, Mobile, Alabama, United States of America; 2 Global Discovery Pathology & Multimodal Imaging, Translation Models Research Platform, Sanofi, Cambridge, Massachusetts, United States of America; 3 Department of Pathobiological Sciences, School of Veterinary Medicine, Louisiana State University, Baton Rouge, Louisiana, United States of America; Boston Children’s Hospital, UNITED STATES OF AMERICA

## Abstract

*Rickettsia parkeri,* an emerging tick-borne pathogen, is transmitted by *Amblyomma maculatum.* Initial bacterial inoculation occurs in the skin via infected tick bite. During blood acquisition, tick salivary factors manipulate host hemostatic and immune responses. Examination of rickettsial pathogenesis and the host response to infection in murine models primarily utilize needle inoculation. As a result, biological variables including transmission dynamics and the influence of vector saliva on the host response at the infection site are undefined. To test the hypothesis that a distinct host cutaneous immune response will be elicited based on the infection status of the tick, a single tick feeding model was developed to assess the transmission and dissemination kinetics of *R. parkeri* from the early stage of tick attachment (1 hour) to late feeding stage (144 hours). Furthermore, changes in the cutaneous immune response were characterized through immunophenotyping, cytokine and chemokine profiling, and histopathological analysis. The data show that transmission of *R. parkeri* from the infected tick to the host occurs within 1 hour-post tick attachment (hpa), with rapid dissemination to lymph nodes and internal tissues. The quantifiable bacterial load in the mouse skin delivered by an infected tick was 9,500 bacteria at 3 hours, increasing to 15,000 and 22,000 rickettsiae at 48 and 144 hours, respectively. Independent of tick infection status, the degree of time-dependent inflammation was similar at 3 and 48 hpa. However, in *R. parkeri*-infected skin at 144 hpa, macrophages were the dominant cell type, with enhanced levels of IFN-γ and TNF-α while IL-10 production was reduced. The data suggest delayed pro-inflammatory activation in response to rickettsial infection when delivered by ticks. This study yields valuable insights into the early events of *Rickettsia* transmission and the host response, providing the basis for the development of preventive strategies against tick-borne rickettsial disease.

## Author summary

Tick-borne diseases have become a significant public health concern in the United States over the past few decades, including spotted fever group *Rickettsia. Rickettsia parkeri* is transmitted to vertebrate hosts by ticks as they take a blood meal. Currently, the minimal time for rickettsial transmission from ticks, the number of rickettsiae inoculated by ticks, and the dissemination pattern to the host tissues, as well as the host response at the tick attachment site, is unknown. Accurate enumeration of rickettsiae delivered by a tick vector and the host response are critical for examining rickettsial pathogenesis and developing disease intervention strategies. Here, we use a natural tick vector and molecular techniques to determine the rickettsial inoculation dose at various timepoints. We observed that *R. parkeri* was transmitted to the host as early as 1 hour after tick feeding, and the inoculation dose by the tick vector is much lower than what is used in needle-inoculation studies. Additionally, we show the impact of tick feeding on the progress of skin inflammation and the host immune response over time at the tick attachment site. Our study provides new insights into rickettsial transmission and dissemination kinetics, the host response, and the interactions between the pathogen with its tick vector and vertebrate host.

## Introduction

*Rickettsia parkeri*, a member of the spotted fever group (SFG) *of Rickettsia*, is a tick-borne, Gram-negative, obligate intracellular bacterium that causes a mild form of spotted fever in humans. Clinically, *R. parkeri*-infected human patients exhibit a combination of non-specific symptoms of febrile illness, including fever, headache, malaise, and myalgia, similar to those seen in other common infectious diseases [1]. A characteristic of *R. parkeri* infection is an “eschar” at the tick inoculation site. In the United States, the Gulf Coast tick, *Amblyomma maculatum*, is the primary vector of *R. parkeri* [1]. Historically, *A. maculatum* distribution was thought to be restricted primarily to the coastal regions of states bordering the Gulf of Mexico and the Southern Atlantic coast. However, in recent decades, its range has expanded northward into the mid-Atlantic states [2]. Rickettsial infection of the tick results in a disseminated infection in which the tick ovary, midgut, and salivary glands harbor rickettsiae [3–5]. The infection of multiple tissues allows for *R. parkeri* to be maintained in the tick life cycle through transovarial transmission, from mother to offspring, and transstadial transmission, sustained in subsequent life cycle stages [5]. Additionally, horizontal transmission of *R. parkeri* to vertebrate hosts is associated with salivary gland infection [3,4]. However, during tick feeding, rickettsiae are deposited with tick saliva at the tick attachment site. The transmission events facilitating SFG *Rickettsia* delivery to the vertebrate host are poorly understood.

Tick-associated variables governing pathogen transmission include the tick species and life cycle stage, tick feeding behavior, and feeding duration. For some tick-borne pathogens, a required period for metabolic reactivation, replication, or migration to the salivary glands is necessary for transmission via saliva to vertebrate hosts [6]. Transmission of tick-borne viruses is observed in as few as 15 minutes of tick attachment [7]. Comparatively, *Anaplasma* spp. and *Ehrlichia* spp. require 24 and 36 hours of feeding, respectively, for bacteria to be transmitted from tick to host [8–10]. The Lyme disease agent, *Borrelia burgdorferi*, is transmitted by ticks approximately 48 hours after tick attachment [11,12]. Seminal studies by H.T. Ricketts demonstrated that an actively feeding *Rickettsia*-infected tick can transmit enough agent to induce disease after 12 hours [13]. For *R. parkeri*, transmission from infected ticks to vertebrate hosts occurs within 2 days of tick feeding, the earliest timepoint assessed [3]. However, cases of *R. parkeri* rickettsiosis in humans were reported with a tick attachment of less than 8 hours [14,15]. While the transmission kinetics for several tick-borne pathogens have been examined, the minimal tick attachment time required for transmission of SFG *Rickettsia* remains unknown.

The process of bloodmeal acquisition by ticks is complicated. Adult ticks can be attached to a host and feed for up to two weeks, depending on tick species [16,17]. To facilitate successful hematophagy, ticks secrete a complex array of salivary molecules into the host, which modulate host hemostatic and defensive responses [18–20]. These actions are critical for maintaining blood flow and preventing host rejection. Conversely, the host mounts countermeasures by activating inflammatory pathways, hemostatic processes, and the complement cascade in response to tick salivary components [18–22]. During the early period of tick feeding, humans develop a strong innate immune response, observed by the infiltration of macrophages, neutrophils, and dendritic cells (DCs), with the upregulation of interleukin (IL)-1β, IL-5, C-C motif chemokine ligand (CCL) 2, CCL3, C-X-C motif ligand (CXCL) 1, and CXCL8 mRNA levels [23]. Equivalent numbers of inflammatory cellular infiltrates are also noted at the tick bite site in murine models [24–26]. In addition to facilitating feeding, bioactive tick salivary factors are recognized to play a role in pathogen infection. Likewise, bacterial pathogens, including *R. parkeri*, manipulate mammalian host molecular processes to facilitate infection [19,27]*.* When compared with needle inoculation, tick feeding or tick saliva enhances *R. parkeri* infection and dissemination, and induces a distinct cellular immune response compared to *R. parkeri* alone [4,27,28]. Nonetheless, while the importance of tick saliva in modulating the host response at the tick bite site is recognized, previous studies have not captured rickettsial transmission in the context of tick-host interactions.

Vertebrate skin represents a key environmental interface acting as a physical and immune barrier, providing the first line of defense to infection [29–31]. *In vitro* and animal model studies implicate numerous elements of the immune system, including endothelial cells, macrophages, natural killer (NK) cells, DCs, CD4/8 T-cells, antibodies, and cytokines and chemokines in the host response to rickettsial infection. During early infection (2–6 days), increases in NK cell activity and interferon (IFN)-γ serum levels have been reported in *Rickettsia conorii*-infected C3H/HeN mice [32]. The host cellular response is important for controlling infection, as depletion of NK cells enhances the susceptibility of mice to rickettsial infection [32]. Additionally, T-cells play a dominant role in defense against rickettsial infection, with depletion of CD8^+^ T cells leading to enhanced susceptibility of C3H/HeN mice [33,34]. Further, elevated cytotoxic activity and release of IFN-γ by CD8^+^ T-cells were observed in SFG *Rickettsia*-infected mice [33,35]. Although numerous studies have examined the host immune response against SFG *Rickettsia*, the utilization of needle inoculation to introduce SFG *Rickettsia* constructs a gap in understanding the tripartite interaction between tick-*Rickettsia*-host at the skin interface.

Compared to needle inoculation, the natural route of rickettsial inoculation by tick results in a lower dose over an extended period of time [3]. Thus, needle inoculation fails to capture the complexity of the transmission process and interaction between the tick and the host’s immune response. We hypothesize that a distinct host cutaneous immune response will be elicited based on the infection status of the tick. In the current study, a single infected adult tick feeding bioassay was developed to recapitulate the tick encounter scenario that most commonly occurs in human exposure [36]. This study provides temporal transmission and dissemination kinetics and evaluates the immune landscape at the site of *R. parkeri* inoculation when delivered by the natural tick vector. The multi-faceted investigation of the complex tick-*Rickettsia*-host interaction serves as an experimental platform to study other tick-borne rickettsial diseases and ultimately allows for studies to decipher the mechanisms of rickettsial disease.

## Results

### Transmission and dissemination of *R. parkeri* occurs early in the tick feeding event

The duration of time period between bacterial pathogen delivery from tick to vertebrate host varies widely between different bacterial genera, as well as species and strains [37]. For SFG *Rickettsia*, the earliest tick transmission examined was 4–8 hours, which was sufficient for *Dermacentor variabilis* to transmit *R. rickettsii* to naïve Guinea pigs and induce disease [38]. The study design employed paired male and female ticks on one animal, thus the transmission potential of an individual tick was not examined. To address this, we have examined earlier timepoints and used individual ticks, a system more consistent with observations in human tick encounter scenarios [36]. In the current study, we utilized a single, female *R. parkeri*-infected *A. maculatum* model and allowed the tick to feed on C3H/HeN mice for 1, 3, 6, 18, 24, 48, and 144 hours (Fig 1). No clinical signs of disease, including lethargy, ruffled fur, hunched posture, or reduced appetite, were observed in mice over the course of *R. parkeri*-infected tick feeding. Transmission of *R. parkeri* occurred as early as 1 hour post-tick attachment (hpa), with subsequent increase in both bacterial load (rickettsiae/host cells) and number of positive samples at 3, 48 and 144 hpa (Fig 2). Trace amounts of rickettsial DNA, below the quantification threshold for quantitative PCR (qPCR; Ct > 35 < 40 cycles), were detected in 87.5% (7/8) mice at 1 hpa. For these seven trace-positive samples, sequence analyses confirmed a 100% identity with *R. parkeri* (Genbank accession number: CP003341.1), suggesting that low-level transmission from tick to host occurred as early as 1 hour after tick attachment. Subsequent increase in quantifiable bacterial burden was observed at 3 (6.4-fold), 48 (13.3-fold), and 144 (30.7-fold) hpa. The quantifiable inoculation dose delivered by an infected tick was 9,500 bacteria at 3 hours, increasing to 15,000 and 22,000 rickettsiae at 48 and 144 hours, respectively (Table 1). Notably, skin was the only tissue type that harbored rickettsial DNA at all timepoints assessed**.**

**Table 1:** Quantifiable rickettsial load at the tick attachment site (6-mm skin sample) and in pooled internal tissues (heart, kidney, spleen, lung, liver, and lymph nodes).

Timepoint (hour-post tick attachment)	Skin bacterial load (Mean ± SEM)	Pooled internal tissue bacterial load (Mean ± SEM)
1	2,485	1,962 ± 848
3	9,489 ± 3,856	19,092 ± 3,835
6	1,996	Unquantifiable
18	7,730 ± 5,584	1,965 ± 671
24	1,946	17,635 ± 4,483
48	15,105 ± 4,699	776 ± 272
144	22,004 ± 5,600	11,332 ± 4,180

**Fig 1:**
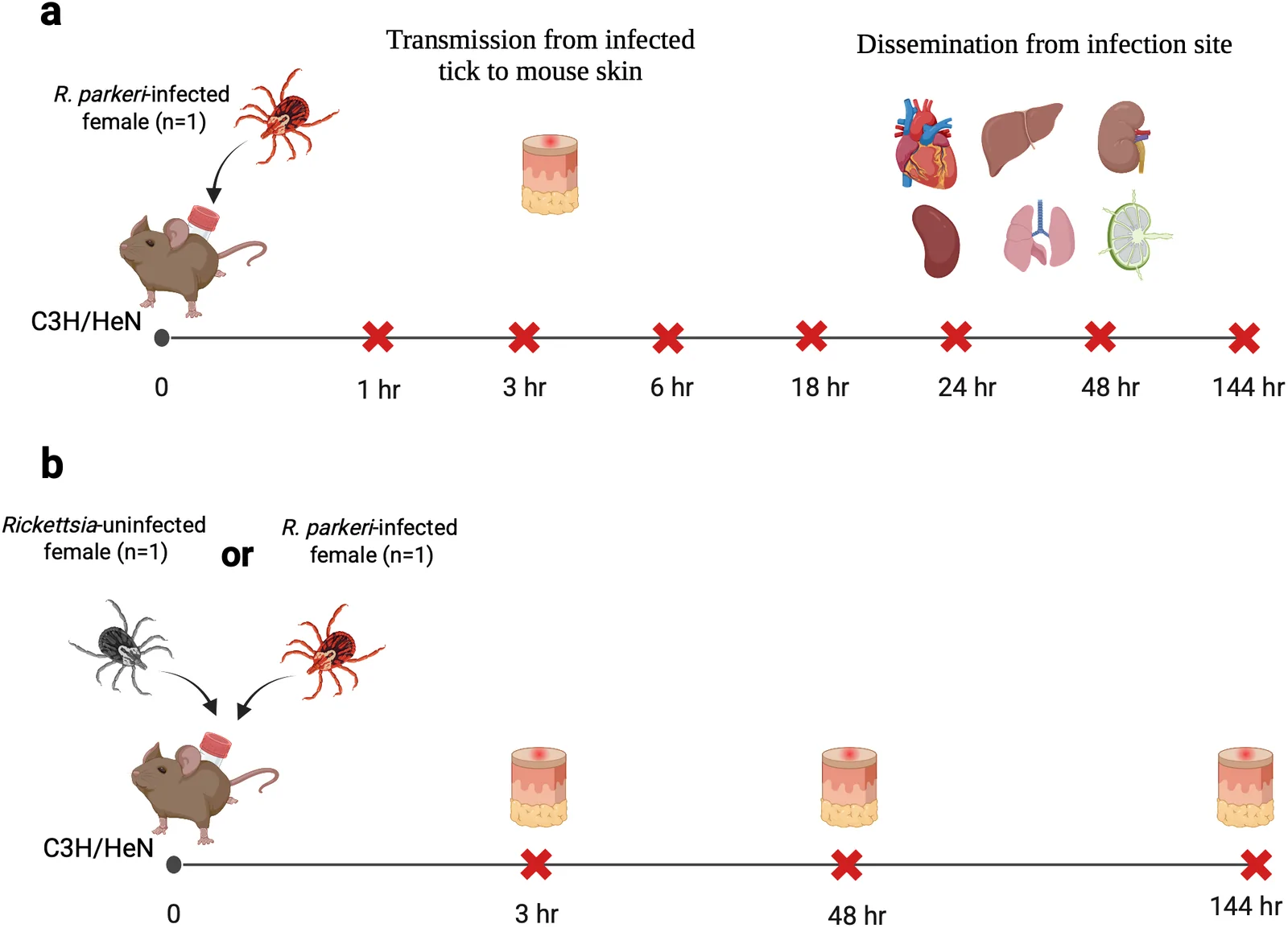
Schematic representation of the experimental approach and sample collection timeline. **(a)** Transmission and dissemination bioassay, a single female *R. parkeri*-infected tick was fed on mice for 1, 3, 6, 18, 24, 48, or 144 hours post-tick attachment. Mice (8 mice per timepoint) were euthanized at each timepoint (red cross). Skin at the tick attachment site and other tissues, including heart, liver, spleen, lung, kidney, and lymph nodes (cervical, axillary, brachial, and inguinal) were collected to assess dissemination and determine bacterial load by qPCR. **(b)** A single *Rickettsia*-uninfected or *R. parkeri*-infected female tick was fed on mice for 3, 48, or 144 hours post-tick attachment. Skin at tick attachment site was harvested for flow cytometry, histopathology analysis, and cytokine/chemokine expression profiling assay. Graphic created in BioRender.com.

**Fig 2:**
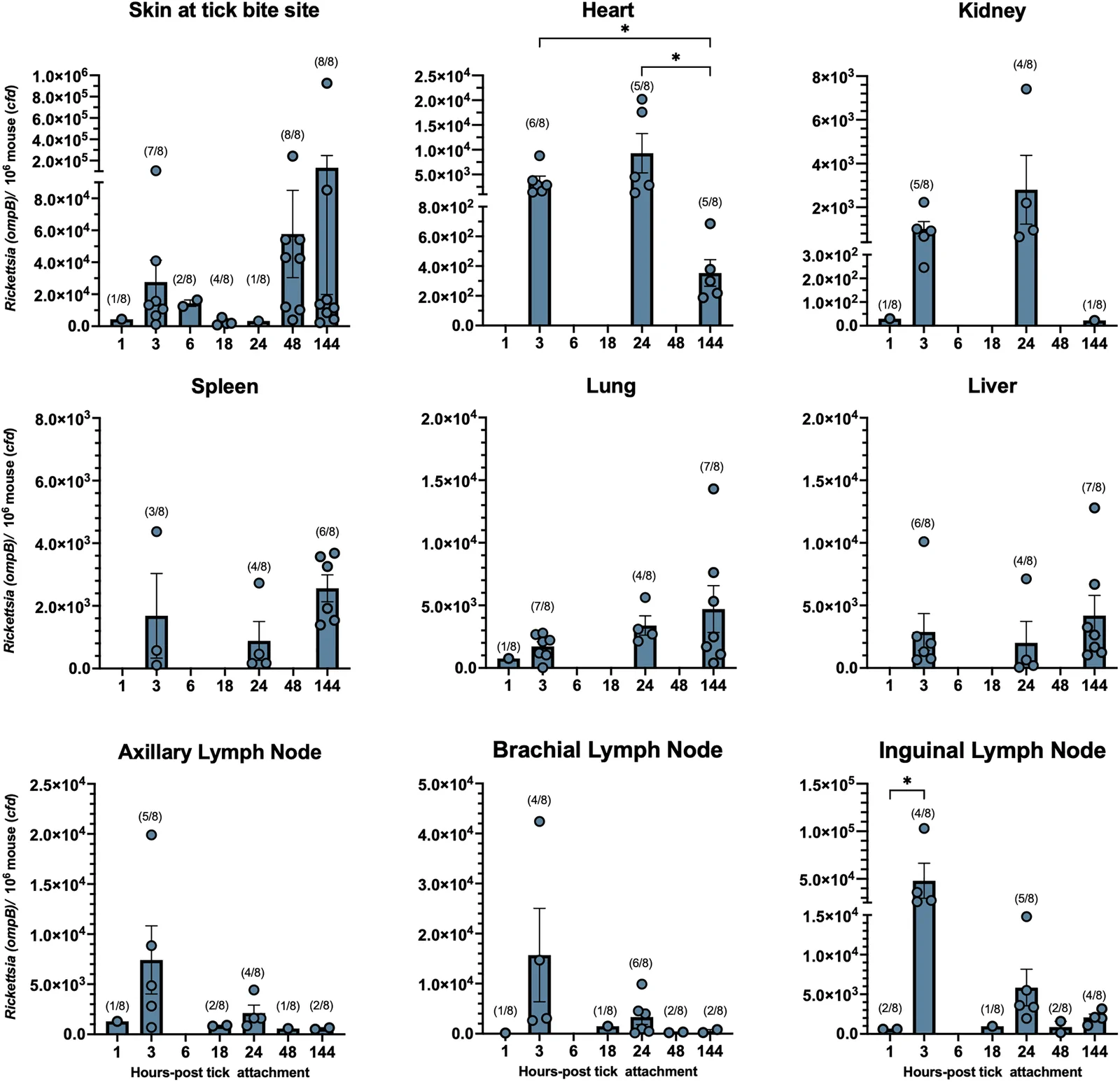
Rickettsial load in mouse tissue samples by a single infected tick feeding. Mice were infested with *R. parkeri*-infected ticks, and tissues were collected for enumeration by species-specific qPCR at specific time points. Each circle represents a tissue sample from an individual mouse (n = 8 mice/time point), and the prevalence of positive, quantifiable samples by qPCR is shown (positive/total). The data are represented as the mean ± SEM. Significance was assessed by one-way ANOVA followed by Holm-Sidak’s multiple comparison test; *p < 0.05.

Early rickettsial dissemination from the inoculation site to distal tissues was also observed at 1 hpa in the lung, kidney, and all tested lymph nodes. At 3 hpa, higher prevalence of positive samples was observed in all tissue samples with greater rickettsial burden in heart, kidney, spleen, lung and liver samples (Fig 2). Similar observations in rickettsial load were observed in the lung, kidney, and heart at 24 hpa. At 144 hpa, rickettsial DNA was detected in all tissues. The heart showed significant decrease in rickettsial load at 144 hpa when compared to 3 hpa, with similar trend also observed in the kidney. At sites other than the skin, when combining the total rickettsial number across all tissue samples, the rickettsial load increased from 1 to 3 hpa, ranging from ~2,000 to ~19,000 rickettsiae, before declining from 6 to 18 hpa. The rickettsial burden peaked at 24 hpa with ~17,600 rickettsiae and subsequently decreased to ~770 and ~11,000 rickettsiae at 48 and 144 hpa, respectively (Table 1). Rickettsial dissemination to the lymph nodes was observed at 1 hpa with significant increase in rickettsial load at 3 hpa in the inguinal lymph node (Fig 2). Furthermore, with the exception of 6 hpa, rickettsial DNA was consistently detected in the lymph nodes throughout the duration of tick attachment. Despite detection in the skin, no rickettsial DNA was detected in the non-lymphoid tissue samples at 6, 18, and 48 hpa. Altogether, the data suggest that one hour of tick attachment is sufficient for *R. parkeri* transmission, with widespread dissemination of rickettsial DNA in vertebrate hosts occurring by 3 hpa.

### Tick feeding alone drives cellular infiltrate to the attachment site

In the transmission and dissemination assessment, the initial quantifiable event of *R. parkeri* inoculation by >50% of the ticks was observed at 3 hpa, with bacterial loads subsequently increasing at 48 and 144 hpa. Thus, these specific timepoints were chosen to examine the direct impact of *R. parkeri* on mouse cutaneous cellular profiles (Fig 1). The single tick model, utilizing either an uninfected (UNT) or *R. parkeri*-infected (RPT) tick, was employed to evaluate skin leukocyte populations at the attachment site. We first compared tick feeding groups to the untouched control (UNC) mouse skin. Regardless of tick infection status, significant increase in CD45^+^ cells at the tick bite site was observed compared with the UNC group except at 3 hpa in the RPT group (S1 Fig). The increases were 3.3-fold, 9.4-fold, and 90.1-fold in the UNT group, and 1.9-fold, 8.8-fold, and 105.1-fold in RPT at 3, 48, and 144 hpa, respectively, suggesting that tick feeding alone significantly impacts the cutaneous immune cell profile.

In the context of infection, we next compared the UNT and RPT groups; no significant differences were observed across all cell types at each timepoint (Fig 3). However, at 3 hpa, the RPT group showed reduction in monocytes and neutrophils compared to the UNT group. When comparing within each tick feeding group over time, monocytes, macrophages, T-cells, αβT-cells, γδT-cells, and DCs showed statistically significant increase in cell number from 3 to 144 hpa and from 48 to 144 hpa. For both groups, no significant change in cellular infiltrate was observed from 3 to 48 hpa. Specifically, in the UNT group, comparisons from 3 to 48 and 144 hpa showed increases in monocytes (1.2-fold and 4.9-fold), macrophages (5.3-fold and 52.0-fold), T-cells (4.3-fold and 27.2-fold), αβT-cells (4.1-fold and 64.9-fold), γδT-cells (1.7-fold and 8.9-fold), DCs (1.5-fold and 6.2-fold), and neutrophils (1.7-fold and 53.6-fold). Similarly, in the RPT group, the increases from 3 to 48 and 144 hpa encompassed monocytes (4.0-fold and 21.5-fold), macrophages (6.0-fold and 75.6-fold), T-cells (5.4-fold and 39.3-fold), αβT-cells (11.9-fold and 308-fold), γδT-cells (1.6-fold and 5.7-fold), DCs (2.1-fold and 10.6-fold), and neutrophils (8.7-fold and 216.8-fold). Despite greater fold increases in monocytes, macrophages, T-cells, αβT-cells, DCs and, neutrophils in the RPT group, no significant differences were observed between the two tick feeding groups at any timepoint. These data suggest that the host infiltrating cellular response is primarily directed toward tick saliva rather than rickettsiae.

**Fig 3:**
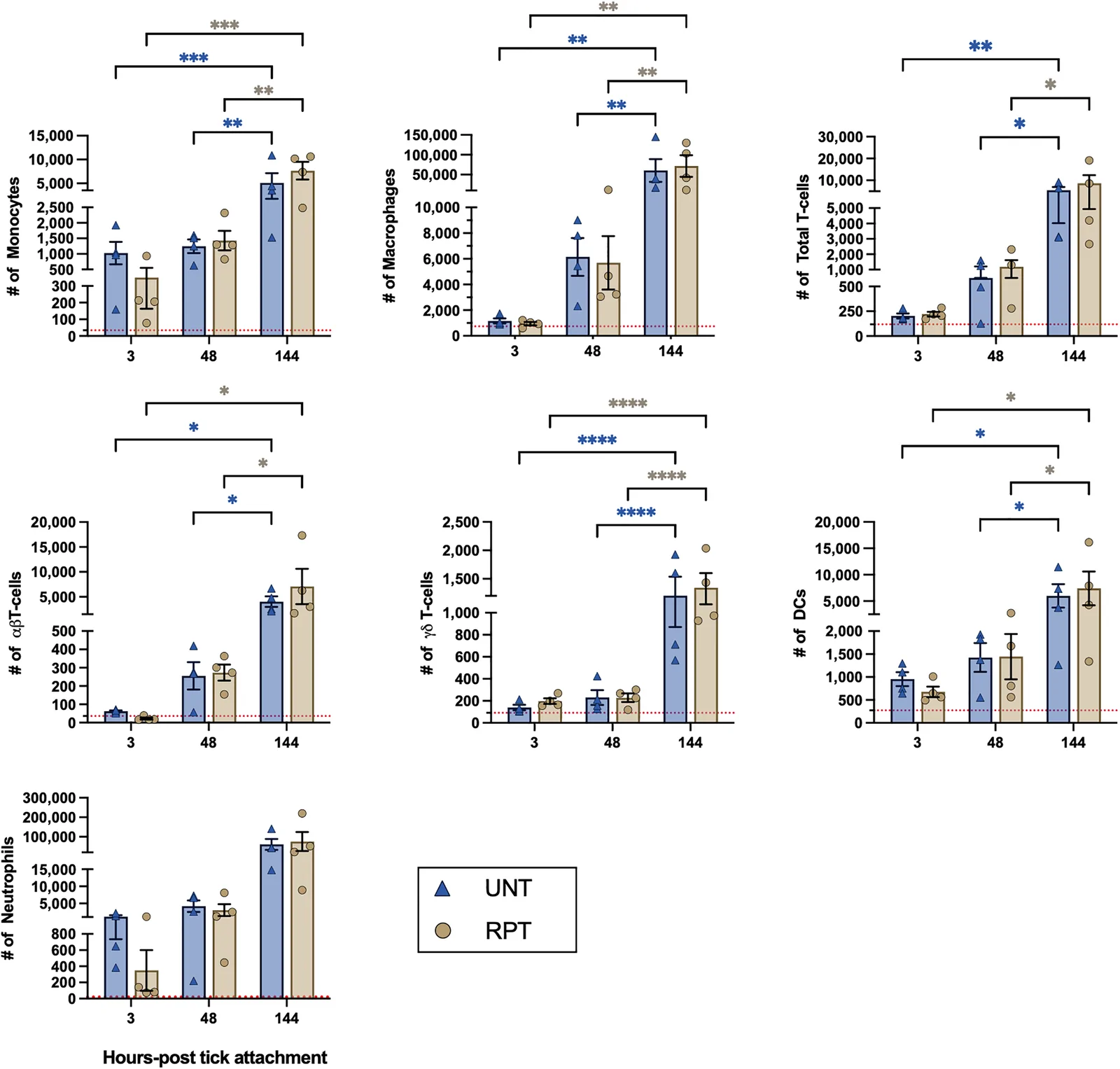
Cellular infiltrate at the tick attachment site. A single *Rickettsia*-uninfected (UNT) or *R. parkeri*-infected (RPT) was fed on mice for 3, 48, and 144 hours. Skin samples were collected to evaluate the cellular response at the tick attachment site. Each symbol (triangle and circle) represents a pool of two mouse skin samples from the tick attachment site, a total of 8 mice/timepoint/group. The red dashed line indicates the mean number (baseline) of cells from the untouched control mouse skin. The data are represented as the mean ± SEM. Statistical significance was assessed using two-way ANOVA followed by Tukey’s multiple-comparison test, with a single-pool variance. (*p < 0.05; **p < 0.01; ***p < 0.001; ****p < 0.0001. At each timepoint, no differences were identified between UNT and RPT groups. Asterisks denote a significant difference when comparing across time for the UNT (blue) and the RPT (brown).

### Tick feeding triggers a time-dependent inflammatory response, coinciding with macrophages as the predominant cell type at the infected tick attachment site

Single tick feeding induced a time-dependent inflammatory response, ranging from none to minimal at 3 hpa to marked by 144 hpa (Fig 4). At 3 and 48 hpa, the degree of inflammation was similar between the UNT and RPT groups, although at 48 hpa, UNT samples occasionally showed slightly more inflammation due to small surface crusts at the tick attachment site. By 144 hpa, inflammation was again more pronounced in the UNT group, primarily because of large surface crusts at the tick attachment site, whereas crusts were minimal or absent in the RPT skin. The cellular infiltrate evolved over time: degranulating mast cells predominated at 3 hpa; by 48 hpa, the infiltrate was mixed (including mast cells, eosinophils, lymphocytes, plasma cells, and macrophages); and by 144 hpa, macrophages were a notably prominent component in the RPT group, whereas eosinophils predominated in the UNT group. Additionally, progressive epidermal hyperplasia was observed over time (S2 Fig). The data suggests that *R. parkeri*-infected ticks alter the cellular profile by increasing macrophages at the late timepoint.

**Fig 4:**
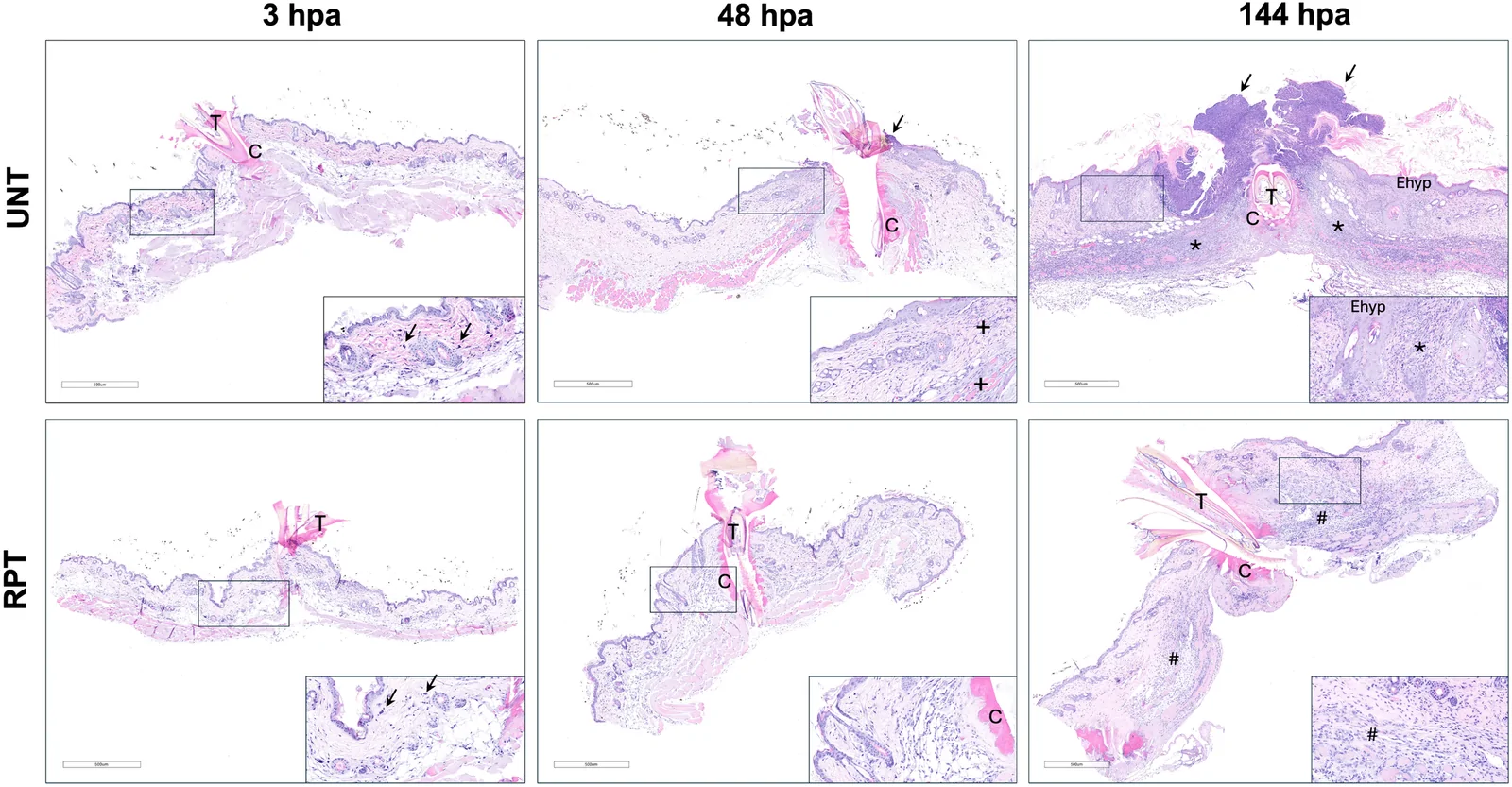
Representative photomicrographs of skin sections at the tick attachment site. A single *Rickettsia*-uninfected (UNT) or *R. parkeri*-infected (RPT) was fed on mice for 3, 48, and 144 hours. Skin samples at the tick attachment site were collected for histopathological evaluation. At 3 hours-post tick attachment (hpa), minimal inflammatory response in both UNT and RPT groups, with degranulating mast cells (arrows) as the predominant cellular component. No marked difference between uninfected and infected sites was observed. At 48 hpa, mixed inflammatory infiltrate including mast cells, eosinophils, lymphocytes, plasma cells, and macrophages were noted. The UNT group occasionally showed slightly more inflammation (plus) associated with small surface crusts at the tick attachment site (arrow). The RPT skin showed a similar degree of inflammation without prominent crusting. At 144 hpa, marked inflammatory response with distinct cellular composition was observed. The UNT skin showed a predominantly eosinophilic infiltrate (asterisks) with large surface crusts (arrow) and notable epidermal hyperplasia (Ehyp) near the feeding site. In contrast, the RPT skin at this late timepoint had macrophage-predominant infiltrate (hashtags) with minimal or absent crusting and less pronounced epidermal changes. C = tick cement and T = tick.

### Tick feeding delays the pro-inflammatory response at the tick attachment site

Next, we evaluated whether tick feeding or *R. parkeri* infection impacts cytokine and chemokine production at the tick attachment site by measuring pro- and anti-inflammatory cytokines and chemokines in the skin at 3, 48, and 144 hpa. First, we sought to determine whether tick feeding alters cytokine and chemokine production at the tick attachment site by comparing the UNT to the UNC group. The data show that there was significant increase in IL-6, IL-17A, IL-17F, GM-CSF, and MCP-1 at the tested timepoints (S3 Fig). Additionally, IL-5 and TNF-α levels were elevated at 3 hpa and at 48 and 144 hpa, respectively. Levels of IFN-γ, IL-13, IL-4, IL-10, and IL-1β were highest at 144 hpa. In contrast, IL-12p70 showed significant reduction at 144 hpa, whereas IL-2 levels remained unchanged at any timepoints.

To determine how *Rickettsia* alters cytokine and chemokine production, we compared skin samples from the tick attachment site between the UNT and RPT groups. Several cytokines exhibited significant variations across the feeding timepoints. IFN-γ levels were markedly elevated in mice exposed to *R. parkeri*-infected ticks at 48 hpa and remained elevated at 144 hpa. The concentrations of TNF-α, MCP-1, and GM-CSF were significantly increased only at the later timepoint (Fig 5). Conversely, IL-10 and IL-12p70 levels were consistently reduced in the RPT group compared to the UNT group throughout feeding, with significant differences observed at 144 hpa. Additionally, in animals exposed to RPT, IL-17F levels were significantly reduced at 48 and 144 hpa.

**Fig 5:**
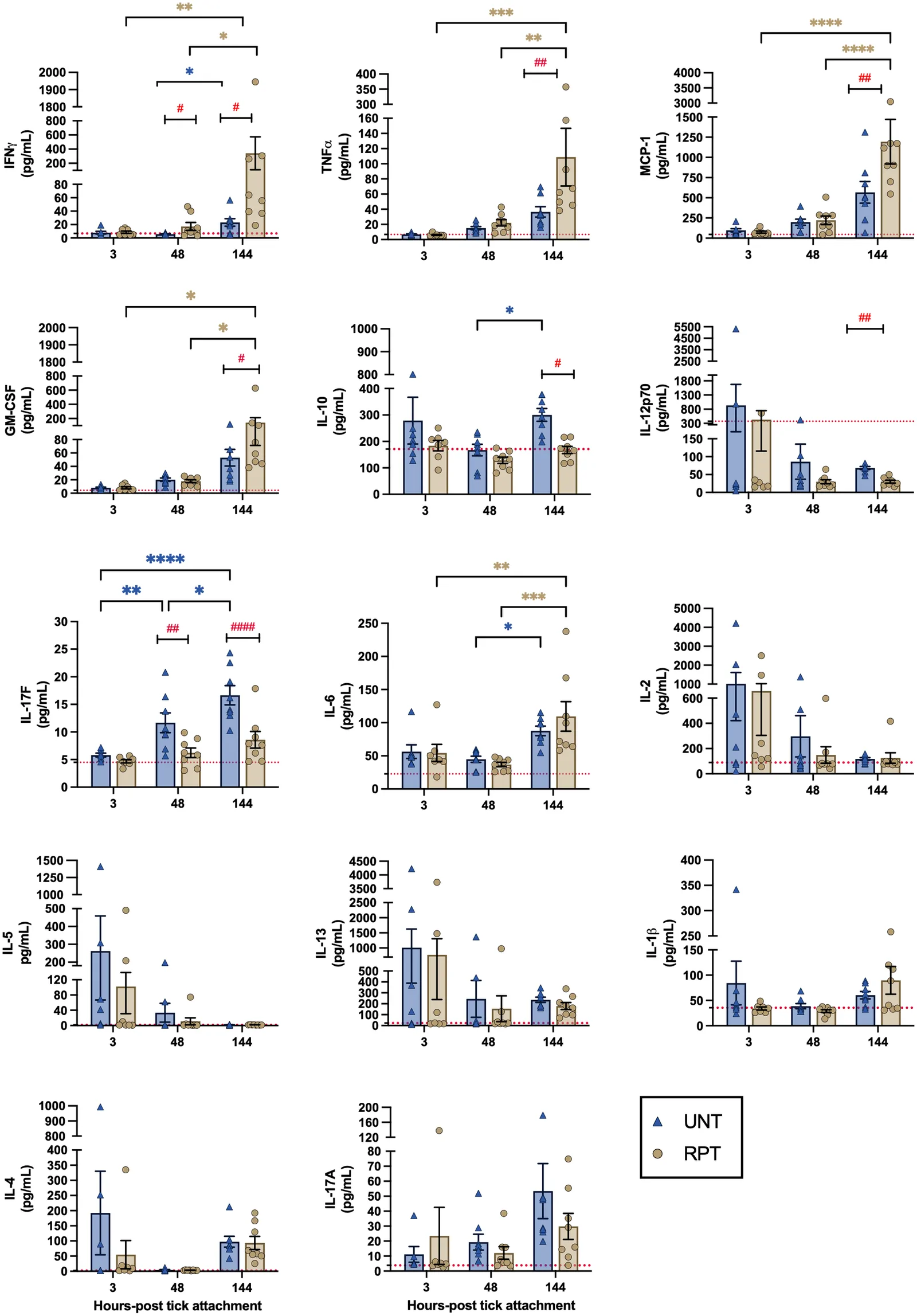
Cytokine and chemokine expression at the tick attachment site. A single *Rickettsia*-uninfected (UNT) or *R. parkeri*-infected (RPT) was fed on mice for 3, 48, and 144 hours. Skin samples were collected to evaluate the cytokine and chemokine expression at the tick attachment site. Each symbol (triangle and circle) represents a single mouse skin sample from the tick attachment site, with a total of 8 mice/timepoint/group. The red dashed line indicates the mean concentration (baseline) of cytokine or chemokine expression from the untouched control mouse skin. The data are represented as the mean ± SEM. Statistical significance was assessed using two-way ANOVA followed by Tukey’s multiple-comparison test, with a single-pool variance (*^, #^*p* < 0.05; **^, ##^*p <* 0.01; ***p < 0.001; ****^, ####^*p <* 0.0001). Asterisks denote a significant difference when comparing across time for the UNT (blue) and the RPT (brown). Pound signs (red) indicate a statistically significant difference when comparing the UNT and RPT.

When comparing cytokine and chemokine production over time, an increase was observed with infection, particularly between 3–144 and 48–144 hpa. The UNT group showed elevated levels of IFN-γ, IL-10, and IL-6 from 48 to 144 hpa. Notably, IL-17F was the only cytokine to exhibit significant increase at all timepoints (Fig 5). In the RPT group, levels of IFN-γ, MCP-1, TNF-α, GM-CSF, and IL-6 increased from 3 to 144 hpa and 48–144 hpa. No significant differences were observed in the levels of other tested cytokines between infection groups (UNT versus RPT) or across the studied timepoints.

## Discussion

Reports of tick-borne rickettsial diseases are increasing [39]. Compounded by the increasing numbers and range of ticks is the recognition of new rickettsial species and isolates that vary in their ability and type of disease they cause [40]. Despite the increase in human infections, little is known about the transmission dynamics that will guide novel intervention strategies. Existing models to assess rickettsial transmission utilize multiple ticks feeding on the individual host [3,41], inconsistent with the probable individual tick bite scenario that is responsible for human exposure [36]. Additionally, studies on rickettsial pathogenesis have relied on needle inoculation models, which fail to capture the complexity of transmission dynamics and do not account for the vector-host immune interaction. Thus, a thorough investigation of the host response to rickettsial infection in the context of tick delivery was lacking. In the current study, we assessed rickettsial infection and dissemination kinetics and employed a comprehensive approach, including immunophenotyping, microscopy, and cytokine and chemokine profiling to investigate the impact of tick feeding on the host response to rickettsial infection.

Tick feeding and pathogen transmission are highly dynamic processes. The current study shows that *R. parkeri* transmission occurs as early as 1 hpa, with subsequent increase in both bacterial load at the tick attachment site and the number of quantifiable transmission events at 3, 48, and 144 hpa. The transmission kinetics fluctuate over the course of tick attachment, congruent with tick feeding biology. Tick feeding involves three stages: attachment to host and feeding lesion formation, slow feeding, and fast feeding [17,42,43]. Within 5–30 minutes of the tick mouthparts penetrating the host skin, a cement compound is deposited as a component of feeding site preparation [42,44,45]. The process of tick cement deposition, which secures the tick’s mouthparts in the vertebrate host skin, can take up to 48 hours. The data in the current study suggest that, in this early phase of site preparation, rickettsiae are secreted along with the components of tick cement. The subsequent variability in bacterial load in the vertebrate host skin corresponds with the nature of tick feeding. Ticks are pool feeders that use a “sip and spit” strategy that alternates between sucking blood, injecting saliva, and resting intervals. The ingestion and salivation cycles typically alternate every 5–20 minutes [46]. Additionally, SFG *Rickettsia* loads in tick salivary glands vary over the course of tick feeding [3,38,47,48]. Thus, the feeding mechanisms of ticks, combined with varying rickettsial load in tick salivary glands, likely contributed to the differences in rickettsiae detected in the skin of the current study. The greatest level of dissemination of *R. parkeri* DNA to internal tissues was observed at 3, 24, and 144 hpa. The early dissemination at 3 hpa suggests that, after inoculation into the skin, bacteria spread rapidly to other tissues. The observed peak dissemination points of rickettsial DNA at 24 and 144 hpa are likely associated with the feeding behavior of the tick and host response to tick feeding. As this study represents the first assessment of rickettsial dissemination when delivered by an individual feeding tick, comparisons to other tick or rickettsial species are not possible. Additionally, the study did not evaluate disseminated infection or bacterial viability in distal tissues but rather assessed disseminated DNA. Future investigations should evaluate rickettsial viability after they leave the tick inoculation site and identify the host cellular response that contributes to rickettsial dissemination.

A challenge in the field is to replicate tick inoculation using needle-based methods. The data from the current study demonstrate that the inoculation *of R. parkeri* into mouse skin via tick feeding results in bacterial doses that are lower than those typically employed in current needle inoculation models, such as intravenous or intraperitoneal injections, which range from 10^6^ to 10^8^ bacteria [41,49–51]. Although we have previously determined that approximately 10^4^ bacteria per microliter were secreted in the tick saliva [3], the partially fed ticks were stimulated to salivate and the cycle of salivation was much faster than the natural tick salivation cycle [52,53]. When examining the rickettsial load when introduced during the tick feeding event, approximately 9,500 bacteria were present after 3 hours of tick attachment, with the bacterial load increasing to about 15,000 and 22,000 rickettsiae in the skin at 48 and 144 hours, respectively. These findings offer a more accurate representation of natural infection dynamics and provide a foundation for improved research approaches. The increase in bacterial load in the skin at later time points could be the result of rickettsial replication at the bite site. However, as *R. parkeri* load in *A. maculatum* saliva increase with feeding time between 2 and 6 days [3], it is likely that more rickettsiae are being delivered to the bite site. Further studies are needed to identify the mechanism, replication versus increased inoculation, that leads to higher rickettsial loads in the host skin at the tick attachment site.

In the skin, the epidermal and dermal layers contain immune cells comprising a complex innate and acquired immune defense network. During tick bloodmeal acquisition, host hemostasis, inflammatory response, and complement cascade are activated against tick salivary molecules [18–22]. In the current study, comparing untouched skin to uninfected tick feeding groups, there was time-dependent increase in inflammatory cellular infiltration at the tick attachment site. All tested cytokines and chemokines were elevated at least once during tick attachment, except for IL-2, which showed no significant change. We observed significant increase in IL-17A, IL-17F, IFN-γ, TNF-α and MCP-1 production in the skin at the tick attachment site. Consistent with observations in human skin, tick feeding upregulates IL-17, IFN-γ, and TNF signaling pathways and elicits a robust local innate immune response, characterized by significant elevation of CCL-2 (MCP-1) [23,54,55]. Because different tick species employ distinct feeding strategies due to variation in mouthpart structure, feeding behavior, cement deposition, and hypostomal depth [44], investigation is warranted into how differences in these feeding mechanisms as well as in saliva composition uniquely shape host immune responses.

The vertebrate host response to rickettsial infection is associated with marked cellular infiltration and dysregulation of cytokines and chemokines. However, as tick feeding elicits a distinct immune profile, the combinatorial effects of tick saliva and rickettsial delivery kinetics result in a dynamic, but overall delayed response. The current study showed that tick feeding masks the early cellular response to rickettsiae up to 48 hours, with significant increase in cellular infiltrate at the tick bite site for all cell types at 144 hpa. Although by flow cytometry no significant differences in cellular numbers were observed between the uninfected and *R. parkeri-*infected groups, there was a greater fold-change increase in monocytes, macrophages, T-cells, DCs, and neutrophils to the infection site from 3 to 144 hours hpa in the infected group. Increase in neutrophil numbers reflects the response to bacterial infection [56,57], and T-cells (CD4 and CD8) have been shown to be critical for controlling rickettsial infection [5,33–35]. Compared to uninfected tick feeding, *R. parkeri*-infected tick feeding was histologically associated with a predominant presence of macrophages at 144 hpa. However, these observations were not supported by flow cytometry data, which showed no significant difference in macrophage numbers at 144 hpa. This discrepancy may be attributable to differences in the analyzed samples, because the histopathological analysis was more precisely localized to the tick attachment site, whereas flow cytometry assessed larger skin samples.

In *R. parkeri*-infected tick feeding samples, the immune cell influx is consistent with the high levels of MCP-1, a chemokine that recruits monocytes to the inflammatory site in the skin. Furthermore, synergistic interactions between MCP-1 and GM-CSF, which was also elevated in hosts exposed to *R. parkeri*-infected ticks, recruits and activates macrophages during inflammation. Using a reductionist approach, previous studies have demonstrated that needle-inoculated *R. parkeri* induces a strong cellular response at the inoculation site by macrophages and neutrophils, but these infiltrates are inhibited by the addition of *A. maculatum* saliva or actual tick feeding at the inoculation site [4,27,28]. The current study is consistent with previous reports, but provides a more nuanced approach to examining the vertebrate host response to SFG *Rickettsia* when inoculated by the tick.

The presence of tick inoculated rickettsiae alters the host cytokine levels at the skin inoculation site when compared to uninfected tick feeding. The elevation of TNF-α and IFN-γ levels in mice exposed to *R. parkeri*-infected ticks at 144 hpa correlates with the greater bacterial load in the skin, indicating the activation of Th1 inflammatory response. The pro-inflammatory cytokines TNF-α and IFN-γ are critical mediators of anti-rickettsial immune responses, as mice depleted of IFN-γ and TNF-α exhibit fatal outcomes due to uncontrolled rickettsial proliferation [58]. Cytokine profiling and mRNA transcript analysis of rickettsial infections in human demonstrate activation of IFN-1 and IFN-γ pathways [59,60]. Supporting the importance of interferon signaling in host defense, intradermal inoculation of *R. parkeri* into the skin of interferon receptor-deficient (double-knockout *Ifnar1*^-/-^; *Ifngr1*^-/-^) mice results in eschar formation at the inoculation site [61]. While delayed, the interferon response by the host is observed in the single tick inoculation bioassay employed in the current study. Increase in IL-6 levels was also observed at 144 hpa in both groups of ticks, with no significant difference between mice exposed to uninfected or *R. parkeri*-infected ticks. These findings corroborate a previous study in which inoculation of *R. parkeri*, with or without tick saliva, increases IFN-γ and IL-6 concentration at the inoculation site [28]. Our previous study demonstrated that intradermal inoculation of *R. parkeri* elicited cellular infiltration and cytokine production as early as 6 and 24 hours post-inoculation [28]. In the current study, we observed that cellular infiltration and cytokine/ chemokine production began to increase at 48 hpa and continued to increase at 144 hours post-inoculation. This delayed response is likely attributable to the lower bacterial dose delivered by the tick vector compared to the higher bacterial inoculum administered via needle. A notable decrease in the anti-inflammatory cytokine IL-10 is observed in mice exposed to *R. parkeri*-infected ticks compared to uninfected ticks, correlating with elevated IFN-γ levels that suppress IL-10 production, leading to a highly polarized, pro-inflammatory immune microenvironment. Consistent with this, reduced IFN-γ levels and increased IL-10 levels in the spleen and serum of *Rickettsia*-infected mice predispose to lethal rickettsial infection [62]. Additionally, significant decreases in IL-12p70 and IL-17F were observed in *R. parkeri*-infected ticks compared to uninfected ticks after the initial 3 hpa assessment, suggesting that *Rickettsia* inhibits production of both cytokines.

In summary, several factors likely influence the successful transmission of, and infection by SFG *Rickettsia*. The model employed in this study did not investigate the relationship between tick inoculation of *R. parkeri* and the formation of an eschar typically observed in *R. parkeri* rickettsioses. Eschars can appear at the tick feeding site after an infected tick has been removed or naturally detaches. In humans, an eschar can form 3–5 days after a tick removal [15,63]. For mice, multiple *R. parkeri*-infected nymphs feeding on an individual C3H/HeN mouse can induce an eschar-like lesion 12 days after ticks attach [41]. Formation of an eschar associated with a single adult tick feeding event has not been described in murine models. In the current study, analysis of the tick attachment site occurred during the feeding process; thus, an eschar was not expected. Future studies can employ this single tick model to determine durations for infected tick attachment and the formation of an eschar. The results of the current study identified an irregular rickettsial inoculation pattern that was neither a bolus nor steady stream of exposure. The dynamic feeding process involving acquisition of blood, concentration of the bloodmeal, and saliva deposition to facilitate feeding likely contributed to the variation in rickettsial loads detected in the skin. Consistent with the variability in rickettsial introduction, the host cutaneous response differed between uninfected ticks and *R. parkeri*-infected ticks. While tick feeding generally delayed the host inflammatory response, at the latest timepoint assessed, distinct differences between the two feeding groups were identifiable. This could be expected as the rickettsial load at the attachment site increased with tick feeding time; however, other factors may also have contributed to the differences. For example, as with other tick-borne pathogens [64,65], the composition of tick saliva changes over time and salivary gland transcripts are altered when ticks are infected with *Rickettsia* [66–68]. The potential that tick-derived molecules capable of modifying the host response are secreted in a differential manner by *Rickettsia*-infected ticks necessities further investigation of the immunomodulatory property of tick saliva, and the interaction between tick and pathogen. The current study provides a framework for the in-depth study of the tick-*Rickettsia*-host relationship, with a broader application to other tick-borne pathogens.

## Materials and methods

### Ticks

Cohorts of *A. maculatum* were maintained in the laboratory, including *R. parkeri-*infected ticks (originated in S. Karim’s laboratory, University of Southern Mississippi) and *Rickettsia*-uninfected ticks (originated from CDC) [4]. During the off-host stage, ticks were held in an environmental chamber at 25–27°C, 90–95% relative humidity, and a 12:12 light:dark cycle. At each generation, prior to use in experiments, a subset of each cohort was tested to verify the presence or absence of *Rickettsia* by qPCR [3,69]. Rickettsial infection status was confirmed by traditional PCR prior to use of ticks in the bioassay, and we observed 100% infection rates of *R. parkeri* in nymph and adult ticks (S4 Fig).

### Mice and tick infestation

C3H/HeN mouse colonies, originating from Charles River Laboratory, were maintained at the University of South Alabama. Mixed sex of mice (male and female 1:1 ratio) aged between six to ten weeks old were utilized in all experiments. For tick infestation, a tick-feeding capsule created from a modified 2 mL screw cap-microcentrifuge was adhered to the dorsum of mice using Kamar glue as previously described [70]. A single female tick from either RPT or UNT cohort was placed into the feeding capsule and allowed to feed for the designated time. After tick placement, the individual tick capsule was open and inspected every 5 mins to verify tick attachment, and the timing was initiated upon observation of the tick’s hypostome embedded into the mouse skin. Mice were housed individually in the ABSL2 animal facility and monitored twice daily for assessment of lethargy, ruffled fur, hunched posture, or reduced appetite as indicators of clinical signs of disease. After designated tick attachment periods, primary and secondary methods of euthanasia that align with the AVMA Guidelines for the Euthanasia of Animals were administered. The number of mice used in each experiment and timepoint are indicated for each bioassay. The UNC samples were obtained from naïve mice with neither tick infestation or capsule attachment and served as baseline control for all studies except the transmission and dissemination assay.

### Ethics statement

All animal procedures were conducted in compliance with protocols approved by the Institutional Animal Care and Use Committee of the University of South Alabama (Protocol Number: 1957072).

### *R. parkeri* transmission and dissemination bioassays

To determine the minimum time required for pathogen inoculation from infected tick to vertebrate host and the dissemination to tissues, single *R. parkeri*-infected female ticks were encapsulated individually on mice and allowed to feed for 1, 3, 6, 18, 24, 48, or 144 hours; 8 mice per timepoint (Fig 1). Timepoints were calculated based on the observed attachment time for each individual tick. At each experimental timepoint, a skin sample was collected from the tick feeding site of an individual mouse using a 6-mm biopsy punch. Additional tissues, including the heart, kidney, spleen, lung, liver, and draining lymph nodes, were also harvested to determine bacterial load by qPCR.

### qPCR for detection of rickettsial DNA

Genomic DNA was extracted from tissue samples using the DNeasy Blood and Tissue Kit (Qiagen) according to the manufacturer’s protocols. Quantification of a rickettsial gene from an individual mouse sample at the selected timepoints were compared by qPCR as previously described [3,50,69,71]. Briefly, rickettsial and mouse DNA recovered from tissue samples was amplified using species-specific primers for *R. parkeri ompB* (outer membrane protein B) [3,4,69] and mouse *cfd* (complement factor D protein) [28,51] genes. Quantification was assessed using dilutions of a linearized plasmid containing the target sequences of *R. parkeri ompB* and mouse *cfd*. Rickettsial loads were calculated as the ratio of *ompB*/*cfd* in order to compensate for variation in tissue sampling size. To verify the trace rickettsial DNA, amplification observed at later cycles (>35 but <40), the PCR products were visualized by electrophoresis on 2% agarose gels. The amplicons were excised from the gel using Zymoclean Gel DNA Recovery Kit (Zymo Research) and then cloned into pCR4-TOPO TA vector (Invitrogen). The plasmids containing PCR products were sequenced (GENEWIZ) with the universal M13 primers and blasted against the NCBI data base [72]. For all qPCR assays, environmental extraction (extraction reagents only) and no template (water) controls were incorporated into the DNA extraction and amplification procedures.

### Flow cytometry

To characterize the immune cell infiltrate, female ticks, either UNT or RPT, were allowed to feed individually for 3, 48, or 144 hours before mice were euthanized and skin samples at tick attachment sites were collected (Fig 1). A total of 8 mice per timepoint were assessed, with two skin samples from the same sex, timepoint, and treatment pooled together to generate a single cell suspension. Two skin samples were pooled to obtain sufficient cell numbers for the analysis. After subcutaneous fat removal, skin samples were transferred with RPMI 1640 medium to gentalMACS C tubes (Miltenyi Biotec) containing digestive enzymes from the Multi Tissue Dissociation Kit1 (Miltenyi Biotec) and incubated 90 mins at 37°C, followed by mechanical dissociation using a gentleMACS Tissue Dissociator. Cell suspensions were filtrated through a 70 µm cell strainer and centrifuged at 300 x *g* for 10 mins at 4°C. Cell pellets were resuspended in the solution containing antibodies to cell surface markers (S1 Table and S5 Fig). After 30 mins incubation at 4°C in the dark, cells were washed and resuspended in PBS + 1% FBS FACs buffer. Data were acquired using the NovoCyte Quanteon Flow cytometer (Agilent), and data were analyzed with FlowJo software version 7 at the University of South Alabama Flow Cytometry Share Resources Laboratories.

### Histopathology

To assess the lesions associated with tick feeding at the site of tick attachment, the RPT and UNT skin samples with the tick intact were collected at 3, 48, or 144 hpa (Fig 1) using a 6-mm biopsy punch and immediately fixed with 10% neutral buffered formalin. After overnight fixation, the skin samples were trimmed, and tick bodies were removed at the basis capitulum, leaving the tick mouthparts embedded in the skin tissue. Tissues containing tick mouthparts were encapsulated in HistoGel (Thermo Scientific) to facilitate proper orientation prior to paraffin-embedding. The specimens then underwent standard histological processing and paraffin embedding (University of South Alabama Health Biobank and Histology Core Facility). Five-micron serial sections were stained with hematoxylin and eosin for histopathological evaluation. Tissue sections were evaluated in a randomized, blinded manner by a board-certified veterinary anatomic pathologist.

### Cytokine and chemokine expression analysis

To evaluate the cytokine and chemokine expression at the site of tick attachment, mouse skin samples were harvested from RPT and UNT groups at 3, 48, or 144 hpa; 8 mice per timepoint per group (Fig 1). Individual skin samples were rinsed with cold PBS, minced into smaller pieces, immediately snap-frozen with dry ice, and stored at -80°C until processed. For processing, samples were lysed in RIPA buffer supplemented with protease/phosphatase inhibitor cocktail plus EDTA (Halts) containing 0.2% w/v type IV collagenase (Worthington Biochemical Corporation) and incubated at 37°C for 1h. Samples were further disassociated using a TissueLyser (QIAGEN) containing a 2-, 3- and 5-mm beads [28]. Following centrifugation at 20,000 x *g* for 10 mins at 4°C, supernatants were collected, and the cytokine/chemokine concentrations were quantified using Mouse Cytokine/Chemokine Focused 14-Plex Discovery Assay Array (Eve Technologies). The analysis was performed using Luminex 200 system (Luminex Corporation) with Bio-Plex Manager software (Bio-Rad Laboratories).

### Statistical analysis

The statistical parameters and significance are reported in the respective figure legends. Statistical analyses were performed using GraphPad PRISM version 10. Data were a combination of two independent experiments (4 mice/timepoint/group/replicate). For the transmission bioassay, statistical analysis was processed using one-way ANOVA followed by Holm-Sidak’s multiple comparison test for bacterial load. For flow cytometry and cytokine/chemokine expression assays, ordinary two-way ANOVA followed by Tukey’s multiple comparison test with a single pool variance was used to analyze the data. Statistical significance was defined by p ≤ 0.05.

## Supporting information

S1 Fig Cellular infiltrate to the tick attachment site. A single *Rickettsia*-uninfected (UNT) or *R. parkeri*-infected (RPT) was fed on mice for 3, 48, and 144 hours. Skin was collected to evaluate the cellular response at the tick attachment site. CD45^+^ cell numbers increased significantly over time in both the UNT and RPT groups compared with the untouched control mouse skin (UNC), except at 3 hpa in the RPT group. Each symbol (diamond, triangle, and circle) represents a pool of two mouse skin samples from the tick attachment site, a total of 8 mice/timepoint/group. The data are represented as the mean ± SEM. Statistical significance was assessed using two-way ANOVA followed by Tukey’s multiple-comparison test, with a single-pool variance (*p < 0.05; **p < 0.01; ****p < 0.0001).
(TIF)

S2 Fig Representative photomicrographs of the untouched control and tick attachment skin sections. Tick feeding increases skin thickness, accompanied by epidermal hyperplasia at the tick attachment site compared with the unremarkable skin of the untouched control (UNC) (a) and *Rickettsia*-uninfected skin (UNT) at 144 hours post-tick attachment (b).
(TIF)

S3 Fig Comparison of cutaneous cytokine and chemokine expression between untouched control and tick attachment skin. Skin samples from the untouched control (UNC) and *Rickettsia*-uninfected tick (UNT) attachment site were collected at 3, 48, and 144 hours post-tick attachment (hpa) to evaluate the cytokine and chemokine expression. Each symbol (diamond and triangle) represents a single mouse skin biopsy from the tick attachment site, with a total of 8 mice/timepoint/group. The data are represented as the mean ± SEM. Statistical significance was assessed using two-way ANOVA followed by Tukey’s multiple-comparison test, with a single-pool variance (*p < 0.05; **p < 0.01; ***p < 0.001).
(ZIP)

S4 Fig PCR confirmation of *R. parkeri* infection in the tick. Adult ticks from *Rickettsia*-uninfected and *R. parkeri*-infected colonies were randomly selected for gDNA extraction. gDNA from an individual tick was amplified using primers specific for *R. parkeri ompB* (a) and *A. maculatum mif* (b) and PCR products were visualized on 2% agarose gel along with GeneRuler 100 bp DNA ladder. Lane 1–7 = gDNA from *Rickettsia*-uninfected tick; lane 8 and 17 = gDNA from positive control sample (*R. parkeri*-infected Vero cells or *A. maculatum* nymph); lane 9 = DNA ladder; lane 10–16 = gDNA from *R. parkeri*-infected tick; lane 18 = blank; lane 19 = no DNA template control, and lane 20 = no DNA template control (gDNA extraction control).
(TIF)

S5 Fig Sequential gating to identify the leukocyte population in mouse skin. Cell debris was excluded, and lymphocytes were selected using forward scatter versus side scatter high gate (R1). Single cells (singlets) were then selected on forward scatter high versus forward scatter area (R2). CD45^+^ cells were selected on a histogram of the singlet cell population (R3). Neutrophils, macrophages, monocytes, T-cells, DCs, αβT-cells and γδT-cells were gated off CD45^+^ cells.
(TIF)

S6 Fig Raw images. (PDF)

S1 Table List of antibodies conjugated with fluorophores used in flow cytometry to detect specific cell type. (DOCX)
