# Supplementary material for: Spotted fever group Rickettsia infection kinetics and cutaneous host response during single tick feeding events
Source: PLoS Pathog. 2026 Sep 28;22(9):e1014626. doi: 10.1371/journal.ppat.1014626 (PMC13638046; doi:10.1371/journal.ppat.1014626)
Supplement: Supplementary file 1 [file ppat.1014626.s006.pdf]

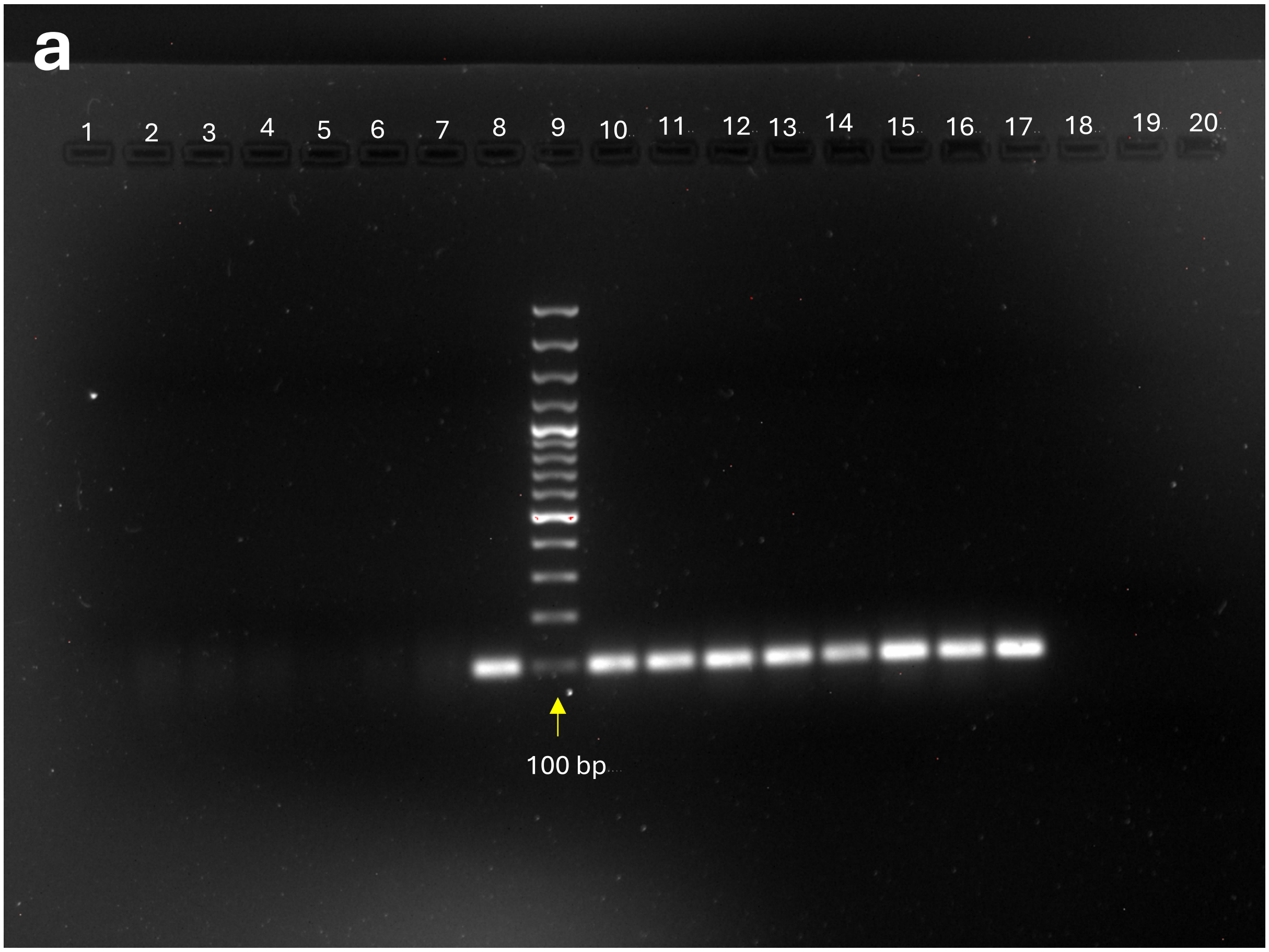
a
1
2
3
4
5
6
7
8
9
10
11
12
13
14
15
16
17
18
19
20
100 bp

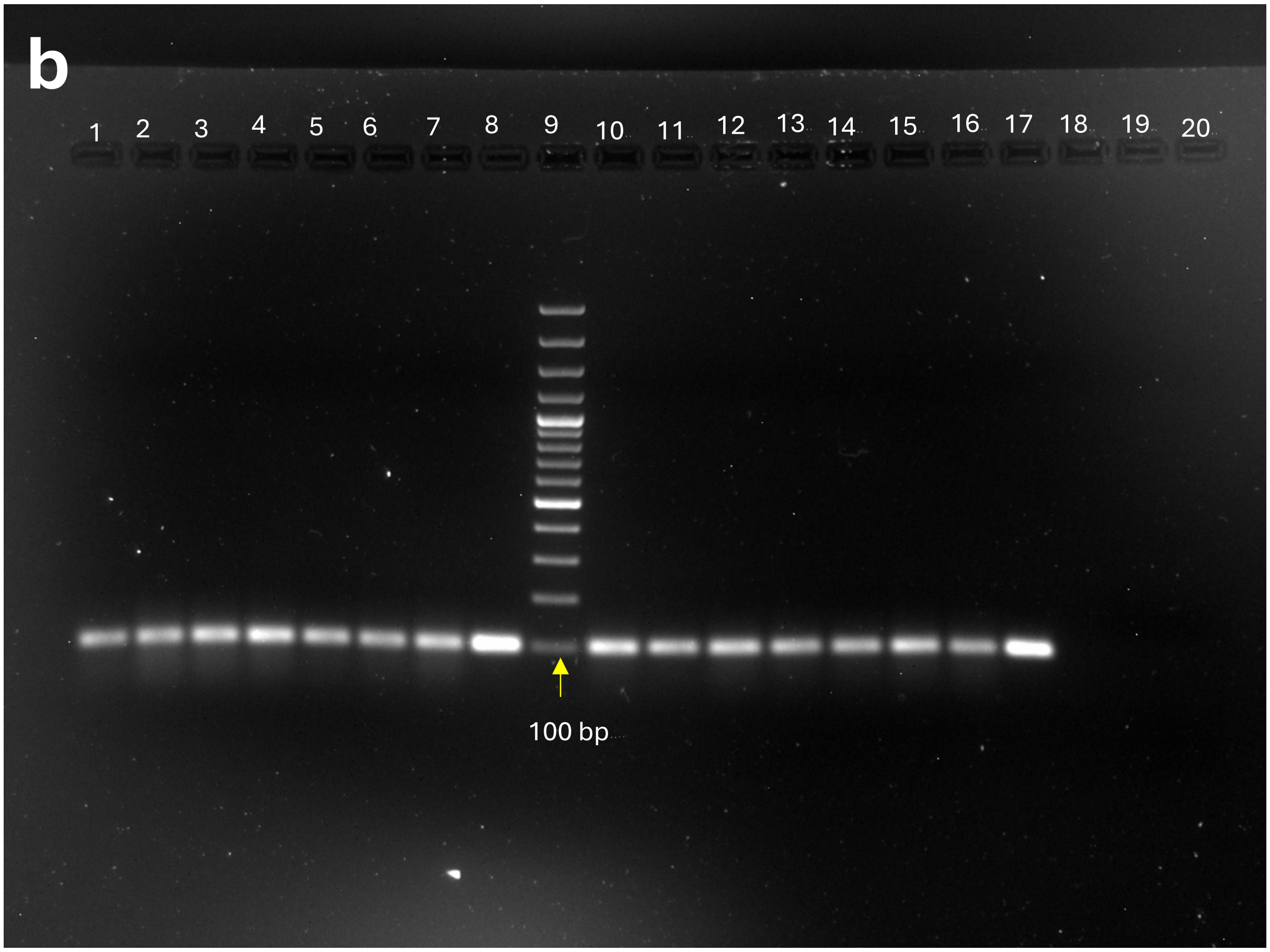

PCR confirmation of *R. parkeri* infection in the tick. Adult ticks from *Rickettsia*-uninfected and *R. parkeri*-infected colonies were randomly selected for gDNA extraction. gDNA from an individual tick was amplified using primers specific for *R. parkeri ompB* **(a)** and *A. maculatum mif* **(b)** and PCR products were visualized on 2% agarose gel stained with SYBR Safe DNA Gel stain. Lane 1-7 = gDNA from *Rickettsia*-uninfected tick; lane 8 and 17 = gDNA from positive control sample (*R. parkeri*-infected Vero cells or *A. maculatum* nymph); lane 9 = GeneRuler 100 bp DNA ladder ; lane 10-16 = gDNA from *R. parkeri*-infected tick; lane 18 = blank; lane 19 = no DNA template control, and lane 20 = no DNA template control (gDNA extraction control).
