# Supplementary material for: Spotted fever group Rickettsia infection kinetics and cutaneous host response during single tick feeding events
Source: PLoS Pathog. 2026 Sep 28;22(9):e1014626. doi: 10.1371/journal.ppat.1014626 (PMC13638046; doi:10.1371/journal.ppat.1014626)
Supplement: Supplementary file 2 [file ppat.1014626.s007.docx]

**S1 Table. List of antibodies used for flow cytometry.**

| Antibody/Target | Fluorophore | Clone | Source |
| --- | --- | --- | --- |
| Anti-mouse CD45 | APC-Cyanine7 | 30-F11 | BioLegend |
| Anti-mouse Ly6G | APC | 1A8-Ly6g | BioLegend |
| Anti-mouse F4/80 | PE-Cyanine7 | BM6 | BioLegend |
| Anti-mouse Ly6c | PerCP-Cyanine5.5 | HK1.4 | BioLegend |
| Anti-mouse CD11c | Brillant Violet 510 | N418 | BioLegend |
| Anti-mouse CD11b | Pacific Blue | M1/70 | BioLegend |
| Anti-mouse GL3 | FITC | GL3 | BioLegend |
| Anti-mouse CD3 | PE-Cyanine5 | 145-2C11 | BioLegend |
| Anti-mouse CD16/32 (Fc block) |  |  | BD Bioscience |
